# Evasins: Tick Salivary Proteins that Inhibit Mammalian Chemokines

**DOI:** 10.1016/j.tibs.2019.10.003

**Published:** 2020-02

**Authors:** Ram Prasad Bhusal, James R.O. Eaton, Sayeeda T. Chowdhury, Christine A. Power, Amanda E.I. Proudfoot, Martin J. Stone, Shoumo Bhattacharya

**Affiliations:** 1 Infection and Immunity Program, Monash Biomedicine Discovery Institute, and Department of Biochemistry and Molecular Biology, Monash University, Clayton, VIC 3800, Australia; 2 Radcliffe Department of Medicine (RDM) Division of Cardiovascular Medicine and Wellcome Trust Centre for Human Genetics, University of Oxford, Roosevelt Drive, Oxford, OX3 7BN, UK; 3 Biopharm Discovery, GlaxoSmithKline, Gunnels Wood Road, Stevenage, Hertfordshire SG1 2NY, UK; 4 Novimmune SA, 14 chemin des Aulx, 1228 Plan les Ouates, Switzerland

**Keywords:** Evasin, chemokine, binding protein, protein family, anti-inflammatory

## Abstract

Ticks are hematophagous arachnids that parasitize mammals and other hosts, feeding on their blood. Ticks secrete numerous salivary factors that enhance host blood flow or suppress the host inflammatory response. The recruitment of leukocytes, a hallmark of inflammation, is regulated by chemokines, which activate chemokine receptors on the leukocytes. Ticks target this process by secreting glycoproteins called Evasins, which bind to chemokines and prevent leukocyte recruitment. This review describes the recent discovery of numerous Evasins produced by ticks, their classification into two structural and functional classes, and the efficacy of Evasins in animal models of inflammatory diseases. The review also proposes a standard nomenclature system for Evasins and discusses the potential of repurposing or engineering Evasins as therapeutic anti-inflammatory agents.

## Highlights

Chemokines are mammalian proteins that are secreted at the sites of tissue insult and stimulate trafficking of leukocytes to the affected tissues, a key component of the inflammatory response.
Evasins are tick salivary glycoproteins that bind to chemokines, thereby suppressing the host inflammatory response, an apparent mechanism to prolong the residence times of ticks on their hosts.
Bioinformatics searches of salivary transcriptomic and genomic sequence databases and yeast surface display screening methods have enabled discovery of Evasins produced by numerous tick species spread across at least three genera.
Two families of Evasins (Classes A and B) have been identified. Evasins from the two families have different 3D structures, different conserved sequence features (including patterns of disulfide bonds), and selectivity for different families of chemokines (CC and CXC, respectively).
Evasins each bind to several (or many) chemokines but different Evasins have distinct selectivities for target chemokines.
Structures, mutational experiments, and sequence comparisons are beginning to reveal the critical elements of Evasins for chemokine recognition.
Evasins show efficacy in animal models of inflammatory diseases, suggesting that either natural Evasins or engineered variants have potential as therapeutic anti-inflammatory agents.

## Chemokine Inhibition as an Anti-inflammatory Strategy

**Inflammation** (see Glossary) is the complex physiological response to tissue injury or infection. A ubiquitous feature of inflamed tissues is the recruitment of **leukocytes**, which function to eliminate pathogens and repair tissue damage but can also perpetuate and amplify the response, leading to chronic **inflammatory disease**. Therefore, selective suppression of leukocyte recruitment is a potential approach to anti-inflammatory therapy.

Leukocyte recruitment in inflammation is regulated by small proteins called **chemokines**, which are secreted at the site of injury or infection and then activate chemokine receptors expressed on the target leukocyte 1, 2 (Box 1). Inhibition of chemokines or receptors could suppress recruitment of the leukocyte subsets expressing the relevant receptors without undesired inhibition of beneficial immune responses. However, effective targeting of specific responses is complicated by the complexity of the chemokine–receptor network, in which most receptors can be activated by several chemokines and most chemokines can activate more than one receptor. Therefore, it would be beneficial to identify or develop agents that can simultaneously target, for example, a group of chemokines that contribute to a particular inflammatory condition.

Box 1 **Chemokines and Chemokine Receptors**

The interactions of chemokines with chemokine receptors regulate the migration of leukocytes to sites of injury or infection and the homeostasis of leukocyte populations in bone marrow and lymphoid organs 60, 61. They can also promote the migration of non-leukocyte cells in development and disease (e.g., cancer cells) and induce other cellular responses such as proliferation and differentiation 11, 62. Figure I shows the complex array of selectivity of human chemokines for human chemokine receptors and the expression of these receptors on different types of leukocytes.

Humans express more than 40 chemokines and many additional gene variants, splice variants, and truncated or otherwise post-translationally modified forms (not shown) [63]. Chemokines are classified, based on the spacing between the two N-terminal Cys residues, into two major families (CC and CXC) and two minor families (CX3CL and XCL) 2, 64. For example, CCL1 is the systematic name for CC chemokine ligand-1, whose previous, nonsystematic name was I-309. CXC chemokines can be further subdivided based on the presence (ELR^+^) or absence (ELR^-^) of the Glu-Leu-Arg sequence, near the N terminus, as this sequence defines selectivity for the neutrophil receptors CXCR1 and CXCR2 [24]. Alternatively, chemokines can be categorized based on their homeostatic versus inflammatory functions [46].

Chemokines collectively target 19 known members of the G protein-coupled receptor family, as well as genetic and splice variants and post-translationally modified forms [63]. Chemokine receptors are classified based on the family of chemokines to which they predominantly bind (e.g., CXCR1 is CXC chemokine receptor-1) and are differentially expressed on various types of leukocytes [1]. In addition, there are also five atypical chemokine receptors (ACKRs), which are expressed on a variety of cell types [1] and are not G protein-coupled but respond to chemokines by recruitment of β-arrestins and internalization, thus removing chemokines from circulation.

**Figure:**
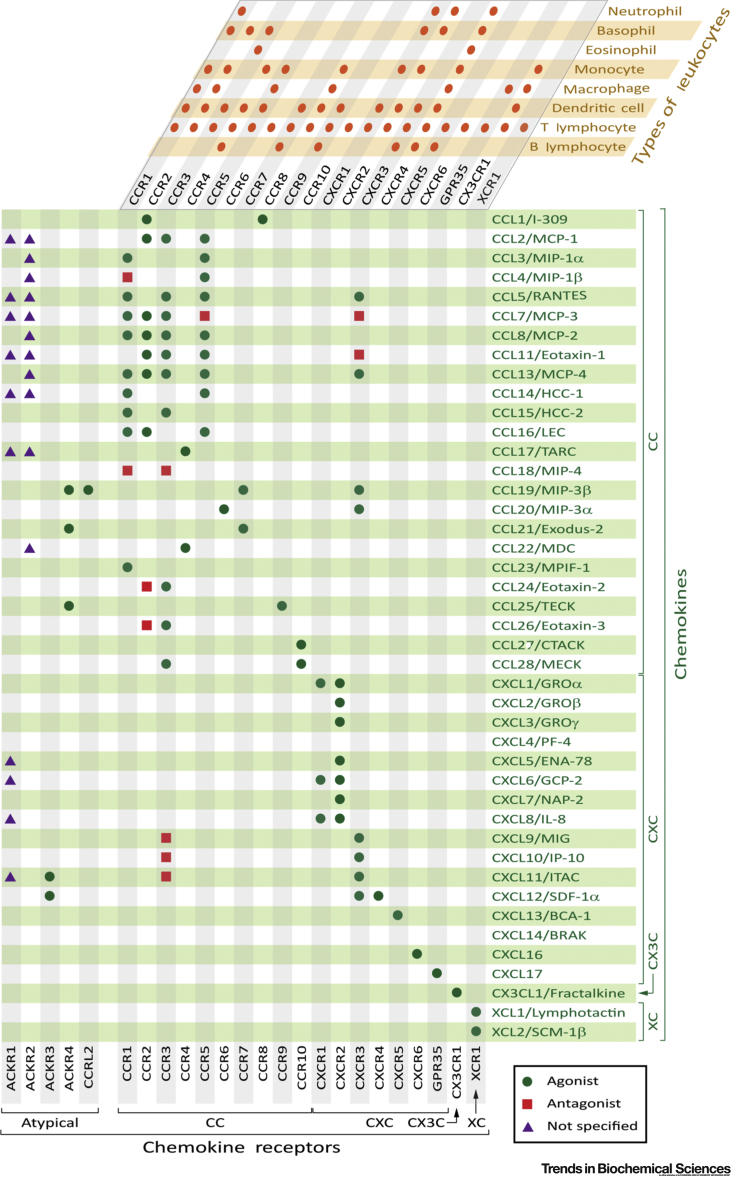
Figure I. Chemokines, Chemokine Receptors, and Leukocytes. The selectivity of human chemokines (listed on right in green) for conventional chemokine receptors (listed at top and bottom in black) and atypical chemokines receptors (listed at bottom left in black) is shown in the green and gray grid. Chemokine–ligand pairs are categorized as listed in the International Union of Basic and Clinical Pharmacology (IUPHAR) Guide to Pharmacology (http://www.guidetopharmacology.org/) (agonists, green circles; antagonists, red squares; not specified, purple triangles). The expression of conventional chemokine receptors on different types of leukocytes (listed top right in orange) is shown in the orange and gray grid; further details, including expression patterns on subtypes of leukocytes (especially T cells) and non-hematopoietic cells, are presented in [1]. Abbreviations for chemokine nonsystematic names: BCA, B cell-attracting chemokine; BRAK, breast- and kidney-expressed chemokine; CTACK, cutaneous T cell-attracting chemokine; ENA, epithelial cell-derived neutrophil activating peptide; GCP, granulocyte chemotactic protein; GRO, growth-regulated oncogene; HCC, hemofiltrate CC chemokine; IL, interleukin; IP, interferon γ-induced protein; ITAC, interferon-inducible T cell α chemoattractant; LEC, liver-expressed chemokine; NAP, neutrophil-activating peptide; MCP, monocyte chemotactic protein; MDC, macrophage-derived chemokine; MECK, mucosae-associated epithelial chemokine; MIG, monokine induced by γ-interferon; MIP, macrophage inflammatory protein; MPIF, myeloid progenitor inhibitory factor 1; PF, platelet factor; RANTES, regulated on activation, normal T cell expressed and secreted; SCM, single cysteine motif; SDF, stromal cell-derived factor; TARC, thymus- and activation-regulated chemokine; TECK, thymus-expressed chemokine.

Considering the roles of chemokines and chemokine receptors in responding to infection, it is understandable that various pathogens and disease vector organisms have evolved mechanisms for inhibiting host chemokines or receptors. These include viruses of the poxvirus and herpesvirus families 3, 4, 5, 6 and the parasitic worm *Schistosoma mansoni* [7]. In addition, hard **ticks**, which are vectors for viral and bacterial pathogens, secrete proteins called **Evasins**, which bind to host chemokines, inhibiting their ability to activate chemokine receptors 8, 9. In the past few years, there has been substantial progress on characterizing these tick Evasins.

There are several possible mechanisms by which chemokine inhibition by Evasins could be advantageous for ticks. Since one effect of inflammation is to make the host aware of the presence of the tick, suppressing inflammation may allow the tick to go undetected and therefore feed longer. In addition, Evasins are likely to reduce immunologically acquired host resistance, thereby increasing tick feeding and survival [10]. Moreover, chemokines are also important for both angiogenesis [11] and fibrotic cutaneous wound healing [12], both of which could be important in defense against ticks.

In this review, we provide a comprehensive account of the current state of knowledge on tick Evasins. Specifically, we describe the initial discovery of Evasins in one tick species, experiments exploring their efficacy in animal models of inflammatory disease, and the subsequent identification of hundreds of potential Evasins from numerous tick species. We describe the classification of Evasins into two protein families and insights into the structural basis of their chemokine recognition and inhibition. To assist future research in this area, we propose a unified nomenclature for tick Evasins. Finally, we discuss the potential of Evasins as clinically useful anti-inflammatory agents.

## Discovery of Tick Evasins from *Rhipicephalus sanguineus*: Evasin-1, -3, and -4

Hematophagous organisms, such as ticks, obtain their nourishment from the blood of their hosts. To do so, they have developed an armory of molecules including anticoagulants, analgesics, and anti-inflammatory molecules that allow them to remain undetected on the host while obtaining their blood feed, sometimes for as long as 2–3 weeks 13, 14, 15, 16, 17. The first evidence of antichemokine activity in ticks came from the observation that salivary gland extracts from several ixodid tick species could neutralize activity of the chemokine CXCL8 18, 19. The same group then showed that tick saliva contains inhibitory activities directed against the chemokines CCL2, CCL3, CCL5, and CCL11 [20]. Importantly, mRNA levels for several of these chemokines are elevated in human skin biopsies from tick bites compared with unaffected skin [21], suggesting that these chemokines are involved in the human reaction to tick bites.

Evidence that the chemokine inhibitory activity was due to discrete molecules was first provided by two experimental approaches in the Proudfoot laboratory: SDS-PAGE analysis of the saliva from the hard tick species *R. sanguineus* (common brown dog tick) following crosslinking to radiolabeled chemokine [9]; and isolation from the saliva by protein chip affinity followed by **mass spectrometric** analysis [8]. Molecular identification of these chemokine-binding proteins by **expression cloning** yielded three novel proteins named Evasin-1, -3, and -4 8, 9. Although the predicted molecular masses of the encoded proteins were 10.5, 7.0, and 12.0 kDa, respectively, these proteins appeared as broad bands of around 30 kDa (Evasin-1 and -3) and 50 kDa (Evasin-4) in the supernatants from HEK293 cells transfected with the tick salivary gland **cDNA library**, because they are all heavily glycosylated. **Glycosylation** may be an important feature to protect the proteins from proteolytic cleavage and immune recognition and thus extend their half-life while the ticks carry out their blood feeds. However, expression of recombinant Evasins has shown that the activity of unglycosylated Evasins was equivalent to the glycosylated forms, indicating that glycosylation is not a prerequisite for chemokine binding and inhibition *in vitro* 8, 9, 22.

Determination of their chemokine selectivity profiles suggested that there were at least two classes of Evasins. Evasin-1 was rather selective, binding to several **CC chemokines** – CCL3, CCL3L1, CCL4, CCL4L1, CCL14, and CCL18. Evasin-4 also bound only CC chemokines but was less selective, binding around 20 CC chemokines [23], although not the monocyte chemoattractants CCL2 or CCL13. However, Evasin-3 bound and inhibited several **ELR**^**+**^ **CXC chemokines** (Box 1) but not CC chemokines 8, 24.

The antichemokine selectivity of Evasins has been tested mostly on human chemokines due to their availability. However, ticks may feed on several different host species, including humans and rodents. The three Evasins identified from *R. sanguineus* inhibit murine as well as human chemokines, as demonstrated in the disease models described below [9], and there is a good correspondence between the selectivity of Evasins for human and mouse chemokines [25]. However, their selectivity for chemokines from other species remains to be thoroughly explored.

## Anti-inflammatory Activity of Evasin-1, -3, and, -4 in Disease Models

Evasins -1, -3, and -4 have all been expressed recombinantly in *Escherichia coli* and/or mammalian cells, enabling evaluation of their therapeutic potential in inflammatory disease models. Evasin-1 reduced neutrophil recruitment induced by CCL3 in a murine peritoneal cell recruitment assay, consistent with the expression of CCR1, a receptor for CCL3, on mouse neutrophils [8]. Similarly, in a mouse model of lung fibrosis induced by administration of bleomycin, Evasin -1 had protective effects and reduced mortality through inhibition of neutrophil infiltration [26]. Evasin-1 also reversed the skin inflammation observed in D6^–/–^ mice in response to 12-O-tetradecanoylphorbol-13-acetate [8], a model previously shown to depend on several inflammatory chemokines [27], suggesting that CCL3 may be a key player in this model. Unfortunately, translation of these results to humans is not straightforward because the cognate receptors for CCL3 (CCR1 and CCR5) are not normally expressed on human neutrophils.

Evasin-3 was also effective in several murine neutrophil-dependent disease models, as expected from its *in vitro* selectivity profile showing that it inhibits ELR^+^ chemokines that activate the receptor CXCR2, which is expressed on neutrophils. Evasin-3 inhibited leukocyte infiltration into the peritoneal cavity in response to CXCL1 [8]. Similarly, Evasin-3 significantly decreased symptoms of antigen-induced arthritis induced by intradermal administration of bovine serum albumin, a highly neutrophil-dependent model [8]. In ischemic reperfusion injury, another neutrophil-mediated model, both Evasin-1 and -3 were protective but Evasin-3 appeared to be more efficacious [8], indicating that the CXCR2 ligands play a predominant role in this model. In contrast, only Evasin-1 and not Evasin-3 was effective in inhibiting the first wave of dendritic cell recruitment to the site of infection with *Leishmania major*, since it is mediated by neutrophil-secreted CCL3 [28].

In line with its broad selectivity profile and inhibitory activity against proinflammatory CC chemokines, Evasin-4 has also been shown to be protective in a number of mouse models, including dextran sulfate-induced colitis 29, 30 and postinfarction myocardial injury and remodeling following left coronary artery permanent ligature [31]. In the latter model, treatment with both Evasin-3 and -4 was associated with beneficial reduction in infarct size and decreases in leukocyte infiltration, reactive oxygen species (ROS) release, and circulating levels of CXCL1 and CCL2. Evasin-4 induced a more potent effect, abrogating the inflammation already observed 1 day after ischemia onset. Although both Evasins failed to significantly improve cardiac function, remodeling, and scar formation, selective inhibition of CC chemokines with Evasin-4 reduced cardiac injury and inflammation and improved survival.

Evasin-3 and -4 have also been compared in a mouse model of acute pancreatitis (and associated lung inflammation) induced by cerulean [32]. Treatment with Evasin-3 decreased neutrophil infiltration, ROS production, and apoptosis in the lung, and reduced neutrophils, macrophage apoptosis, and necrosis in the pancreas. Evasin-4, however, only reduced macrophage content in the lung and did not provide any benefit at pancreas level.

Taken together, the results using these animal models show that Evasins may have therapeutic potential in a variety of inflammatory disease settings but also highlight some of the limitations and potential challenges in translating *in vivo* data from mice to man. Nevertheless, the promise shown by the first three Evasins discovered has provided substantial motivation to identify and develop additional Evasins with suitable chemokine-targeting selectivity for clinical applications.

## Identification of Evasins from Numerous Tick Species

Until recently, only the three *R. sanguineus* Evasins had been characterized. However, searching of expressed sequence tags (ESTs) in public databases and a cDNA library from *R. sanguineus* yielded six additional putative Evasin-1 or -3 homologs 9, 33. Evasin-3-like ESTs have also been identified in I*xodes scapularis*, *Ixodes ricinus*, *and Dermacentor andersoni* [34] and cDNA libraries from *Amblyomma maculatum* and *Amblyomma americanum* also contained at least 18 DNA sequences encoding putative Evasins 35, 36.

In 2017, two laboratories reported combined **bioinformatics** and experimental studies to identify and characterize new Evasin proteins. The Bhattacharya laboratory used psiBLAST to identify over 350 sequences with homology to Evasin-1, -3, or -4 in publicly available transcriptome datasets from prostriate and metastriate ticks [25]. These sequences were then cloned into a **yeast surface display** vector to generate a library of putative Evasins expressed on the surface of yeast, which was then screened against fluorescently labelled CC chemokines to identify chemokine-binding Evasins. Using this technology, 26 Evasin sequences homologous to Evasin-1 and -4 were identified, ten of which were characterized in detail through recombinant expression of these sequences in HEK293 cells. Using a combination of biolayer interferometry and Boyden chamber chemotaxis assays these proteins were shown to bind and neutralize multiple chemokines *in vitro*.

In a parallel study, the Stone laboratory used sequence similarity searches to identify more than 250 sequences homologous to Evasin-1 and -4 in publicly accessible databases and locally obtained transcriptomes [22]. These putative Evasin sequences spanned numerous tick species from the genera *Rhipicephalus*, *Amblyomma*, and *Ixodes*. Of these sequences, nine were successfully expressed using an *E. coli* expression system; eight of these were shown to bind to various CC chemokines and four representative Evasins were demonstrated to inhibit chemokine signaling in cell-based receptor activation assays. Two of the Evasins validated in this study (ACA-01 and RPU-01) [22] were identical to two reported in the parallel study (P974_AMBCA and P467_RHIPU, respectively) [37].

Subsequently, Alenazi *et al.* [37] described independent characterization of one of the CC chemokine-binding Evasins identified by Hayward *et al.* [22] (named P1243 and AAM-02, respectively, by these two groups), as well as a new Evasin (P1156) homologous to Evasin-3 that bound to a number of CXC chemokines. Moreover, by linking these two Evasins together, they created a ‘two warhead’ Evasin capable of binding and inhibiting both CC and CXC chemokines [37]. In addition, Eaton *et al.* reported another new CC chemokine-binding Evasin (P672) homologous to Evasin-1 and -4 [38]. Very recently, Lee *et al.* reported the application of yeast surface display to discover 27 additional Evasins homologous to Evasin-3, most from the same tick species, *I. ricinus* [39].

These studies demonstrate the utility of bioinformatics approaches to identify new Evasins and suggest that many of the >700 Ixodidae (hard tick) species and ∼200 Argasidae (soft tick) species may produce chemokine-inhibitory Evasin proteins. To date, no Evasin homologs have been identified in any non-tick species.

## Classification and Nomenclature of Evasins

Considering that the bioinformatic studies above were based on sequence similarity to *R. sanguineus* Evasin-1, -3, and -4, it is not surprising that the new Evasins (and putative Evasins) identified fall into two sequence families: those with high sequence similarity to *R. sanguineus* Evasin-1 and -4 (defined here as Class A Evasins) and those with high sequence similarity to *R. sanguineus* Evasin-3 (Class B Evasins). In light of the recent increase in the number of known Evasins, we believe it is timely to propose guidelines for the consistent nomenclature of these proteins.

There are several challenges in defining such a system. First, there is no obvious, simple correspondence between the Evasin sequences in one species and those in a related species, suggesting that Evasin sequences have diverged after speciation events. Second, while the Evasins identified to date fall into two sequence families, it is possible that additional, unrelated families will be identified in the future. Third, putative Evasins identified based on sequence similarity to known Evasins are not necessarily functional Evasin proteins, so should not be classified as such until their chemokine-binding properties have been confirmed. Finally, the transcriptome databases for some species contain multiple Evasin-like sequences that are extremely closely related. It remains to be determined whether these represent distinct genes present in all individuals of that species, different alleles of the same gene that vary across the species population, or sequencing or transcriptome assembly errors.

Considering these factors, we propose that each validated (chemokine-binding) Evasin protein sequence should be given a unique name, consisting of the prefix ‘EVA-‘, to designate the protein as an Evasin, followed by the identifier defined in the first publication in which that protein was demonstrated to be a chemokine-binding Evasin. For example, *R. sanguineus* Evasin-1 [9] would be designated simply EVA-1, whereas the protein reported first as P974_AMBCA [25] and subsequently as ACA-01 [22] would be designated EVA-P974; we have dropped the original species-specific suffix ‘_AMBCA’, which is not required for unique identification of this sequence. Tables S1a and S1b in the online supplemental information list the proposed systematic names for all Evasins validated to date, along with their previous designations. We propose that future papers should use these systematic names but also cite the original papers in which any alternative names were used.

## Class A Evasins

### Protein Sequence Features

**Figure 1:**
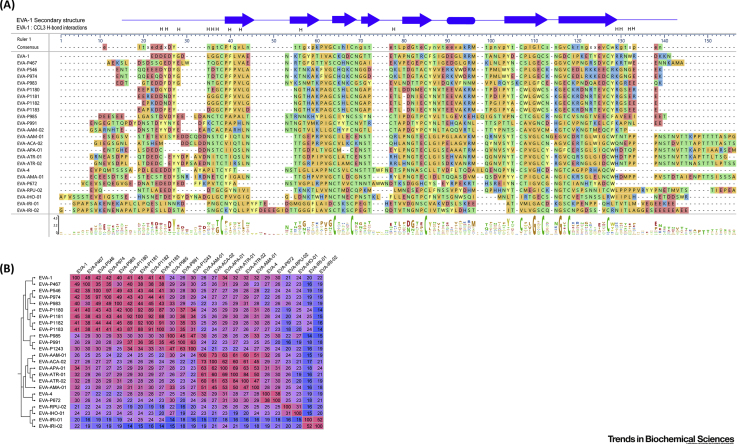
Sequence Alignments, Phylogenetic Tree, and Pairwise Identity Matrix of Class A Evasins. (A) Sequence alignment of all validated Class A Evasins with proposed nomenclature. The consensus sequence (above alignment) and a graphical representation of the amino acid conservation (sequence logo; below alignment) show that eight cysteine residues (green) are conserved (except for two missing Cys residues in the bottom three sequences) and two glycine residues are completely conserved across the family. The secondary structure of EVA-1 [Protein Database (PDB) ID: 3FPR] and EVA-1 residues forming hydrogen bond interactions with CCL3 (in PDB ID: 3FP), analyzed by PDBSum, are presented at the top of the alignment. The alignment was performed using MAFFT, with default parameters, in the program DNASTAR Navigator 15 (DNASTAR, Madison, USA). Amino acid residues are color coded by physicochemical properties (aromatic, light yellow; acidic, medium salmon; basic, medium blue; nonpolar aliphatic, medium orange; polar neutral, medium green). (B) Phylogenetic tree (left side) and pairwise identity matrix of all validated Class A Evasins. Pairwise identities between sequences were calculated using MAFFT and are color coded on a continuous scale from rose (high identity) to blue (low identity). The phylogenetic tree was generated on FigTree v1.4.3 (http://tree.bio.ed.ac.uk/software/figtree/) using the alignment data from MAFFT.

To date, 21 proteins with significant sequence identity to EVA-1 and -4 have been validated as chemokine-binding proteins (Table S1a in the online supplemental information and references therein). Their sequences (89–126 amino acid residues) are aligned in Figure 1A and their phylogenetic tree and pairwise identity matrix are shown in Figure 1B. Pairwise sequence identities among these Evasins range from 15% to 97% (average 31.1%). Nine of these sequences have <30% identity to both EVA-1 and -4, indicating substantial divergence from the earliest identified Evasins.

The sequence alignment highlights several conserved features of the Class A Evasins. Eight cysteine residues that form four intramolecular **disulfide bonds** in the structure of EVA-1 (*vide infra*) are strictly conserved, except for three sequences from the genus *Ixodus*, which are missing the fifth and eighth cysteines, a disulfide pair [22]. The strong conservation of the Cys residues suggests common disulfide-bonded architecture and protein fold. In addition, two glycine residues are completely conserved (*vide infra*).

The Class A Evasin sequences indicate that these proteins are likely to undergo various post-translational modifications. All Evasin sequences contain an N-terminal signal peptide (not shown in Figure 1) that is cleaved off during secretion 8, 9. Twenty-one of the 24 sequences contain at least one tyrosine residue in the N-terminal region in highly acidic sequence environments (2–6 Asp or Glu in the preceding six and following three residues). This type of sequence motif in secreted proteins is highly indicative of a tyrosine sulfation site 40, 41. Moreover, tyrosine sulfation is a common post-translational modification of chemokine receptors, known to enhance their binding affinity and modify their selectivity for cognate chemokines [42]. Thus, although there is no reported experimental evidence on sulfation of Evasins, it is possible that tyrosine sulfation can also modulate chemokine binding by Evasins, an interesting example of evolutionary convergence and molecular mimicry.

Another conserved post-translational modification of Class A Evasins is *N*-glycosylation. The verified sequences each contain several potential *N*-glycosylation sites (Asn-Xaa-Ser or Asn-Xaa-Thr; Xaa indicates any amino acid), consistent with the early characterization studies. Several Class A Evasins also contain potential *O*-glycosylation sites [25].

### Chemokine-Binding Properties

Class A Evasins typically bind to a variety of CC chemokines, inhibiting their receptor binding and activation, and the absolute selectivity of Class A Evasins for CC chemokines over CXC chemokine suggests that they may recognize the CC motif itself. The selectivity of Class A Evasins amongst CC chemokines varies substantially. For example, EVA-1 bound to CCL3, CCL4, and CCL18 with *K*_d_ values below 5 nM, but did not bind detectably to ten other human CC chemokines [9] and EVA-P1183 bound (*K*_d_ <100 nM) to only six of 25 CC chemokines tested [25]. In contrast, EVA-4 bound (*K*_d_ <5 nM) to 17 of 23 chemokines tested [23] and EVA-P991 bound (*K*_d_ <100 nM) to 19 of 25 chemokines tested [25]. The tightest binding reported is an equilibrium dissociation constant (*K*_d_) of ∼1 pM, for binding of EVA-P467 to CCL2 [25]. These selectivity differences raise the possibility of identifying or engineering Evasins for selective recognition of particular chemokines (or groups of chemokines).

The relationships between Evasin sequences and chemokine binding selectivity have not yet been thoroughly explored. However, mutation of EVA-1 residues Phe-14 (near the N terminus) and Asn-88 and Trp-89 (near the C terminus) reduced binding affinity for CCL3 by three- to fourfold 31, 43, consistent with the structure of EVA-1 bound to CCL3 (described below). Similarly, mutation of EVA-4 residues Glu-16 and Tyr-19 reduced CCL5 binding (∼60–80% reduction in a phage display binding experiment), although several mutations near the C terminus of EVA-4 had no effect [31]. The importance of the N-terminal region of Evasins is also supported by a study of a hybrid Evasin consisting of EVA-1 in which the 29 N-terminal amino acid residues were replaced with the N-terminal 44 residues of EVA-P672 [38]. Whereas wild type EVA-1 does not bind to CCL8, both EVA-P672 and the hybrid protein were able to bind and inhibit this chemokine.

### Structure of EVA-1

**Figure 2:**
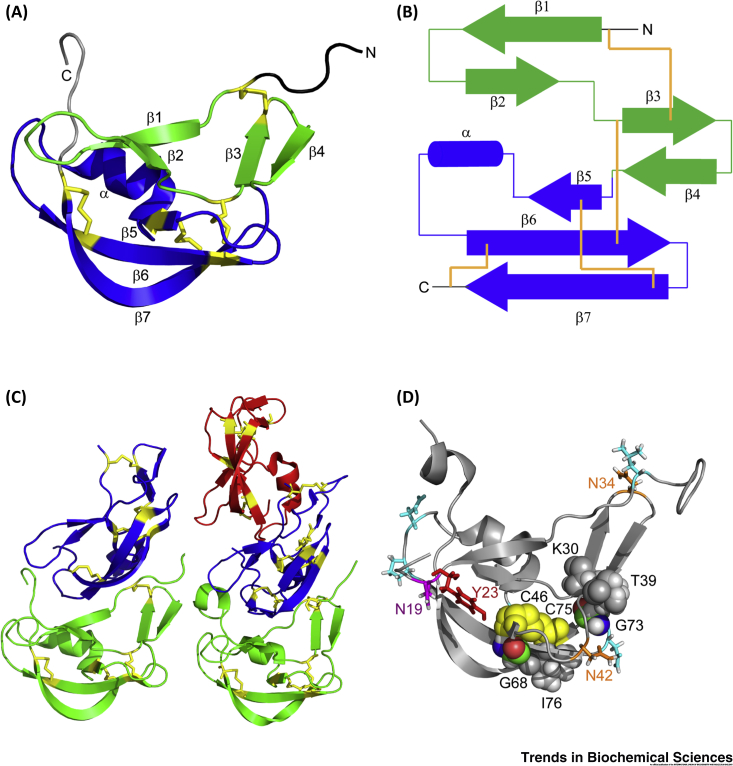
Structure of EVA-1. (A) Ribbon representation of one protomer of nonglycosylated EVA-1 [Protein Database (PDB) ID: 3FPR, resolution 1.70 Å] showing the base of the structure formed of the third β-sheet (β5, β6, and β7 strands) and the α-helix (blue); the first β-sheet (β1 and β2 strands) and second β-sheet (β3 and β4 strands) (green); the N-terminal region (black); the C-terminal region (gray); and cysteine residues and disulfide bonds (sticks) (yellow). (B) Topology diagram corresponding to (A) showing β-strands as arrows and the α-helix as a cylinder, with coloring the same as in (A), except that disulfide bond connectivity is shown as yellow-orange lines. (C) Dimer of nonglycosylated EVA-1 (PDB ID: 3FPR; left) and trimer of glycosylated EVA-1 (PDB ID: 3FPT, resolution 2.70 Å; right). Each protomer is represented in a different color, with cysteine residues and disulfide bonds (sticks) in yellow. (D) Ribbon representation of one protomer of glycosylated EVA-1 (PDB ID: 3FPT) showing confirmed glycosylated residue Asn-19 (magenta sticks); other potential *N*-glycosylation sites Asn-34 and Asn-42 (orange sticks); residues corresponding to predicted glycosylation sites in other Evasins (cyan sticks); and putative sulfation site Tyr-23 (red sticks). The two conserved glycine residues and their interacting residues are shown in space-filling representation with Gly colored by atom type (C, green; H, white; O, red; N, blue), Cys in yellow, and other residues in gray. Gly-68 of EVA-1 is positioned adjacent to the disulfide bond linking strand β5 to strand β7 such that no l-amino acid side chain can be accommodated in this closely packed region of the structure. Gly-73 of EVA-1 is located on the β6–β7 turn with its CH_2_ group packed closely against the side chains of residue Lys-30 (on β3) and Thr-39 (on β4). This interaction may be important to define the relative positions of the β3–β4 sheet and the β5–β6–β7 sheet.

Dias and coworkers reported the crystal structures of both nonglycosylated and glycosylated forms of EVA-1 (Figure 2) [43]. The eight conserved Cys residues define four intramolecular disulfide bonds (Figure 2A,B). However, considering that a subset of Class A Evasins from *Ixodes* tick species lack the Cys residues forming a disulfide between strands β6 and the C-terminal region, this disulfide is probably not essential for structural stability [22]. The flexible N- and C-terminal regions extend from either side of the secondary structural core (Figure 2A) and, together with the first β-sheet, form a concave surface suitable for binding either to another EVA-1 molecule (Figure 2C) or to a chemokine (*vide infra*). All three predicted glycosylation sites of EVA-1, one of which showed electron density for a sugar moiety, and several other residues that correspond to predicted glycosylation sites in other Evasins, are all located on the exterior surface of the structure (Figure 2D), which would readily accommodate glycosylation. The strict conservation of two glycine residues in Class A Evasins can be explained by their locations in spatially restricted regions of the EVA-1 structure.

### Structural Basis of Chemokine Recognition

**Figure 3:**
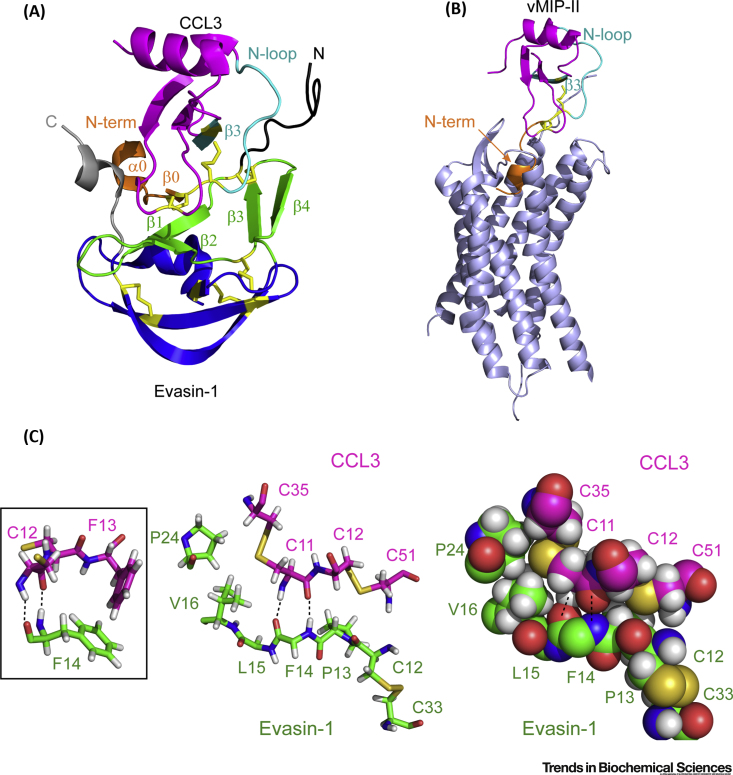
Structure of the Complex between EVA-1 and CCL3. (A) EVA-1 [α-helix and third β-sheet (blue); the first β-sheet and second β-sheet (green); the N-terminal region (black); the C-terminal region (gray); and cysteine residues and disulfide bonds (sticks) (yellow)] bound to CCL3 [magenta but highlighting the N terminus (orange), N-loop and β3 strand (cyan), and conserved cysteine residues with side chain sticks (yellow)] [Protein Database (PDB) ID: 3FPU, 1.9 Å resolution]. (B) The complex (PDB ID: 4RWS) between viral chemokine vMIP-II, colored the same as CCL3 in panel (A), and chemokine receptor CXCR4 (gray); the N-terminal 22 residues of vMIP-II were not defined in this structure. (C) Stick (left) and space-filling (right) representations showing the four Cys residues of CCL3 (magenta carbon backbone and labels) forming two conserved disulfide bonds and the residues of EVA-1 (green carbon backbone and labels) with which they directly interact. The first disulfide bond of EVA-1 (Cys-12 to Cys-33) is also shown. Side chains of EVA-1 Phe-14 and Leu-15 are omitted for clarity. Two hydrogen bonds from CCL3 Cys-11 to EVA-1 Phe-14 are indicated as broken lines; these extend the first β-sheet of EVA-1 by a very short β-strand (β0). The orientation of the CC motif relative to the first β-sheet of EVA-1 is further constrained by hydrophobic interactions of each CCL3 disulfide bond with EVA-1 side chains (Val-16, Pro-24, and Pro-13), which are conserved or substituted by other hydrophobic residues in most other Evasin sequences. The interaction is further stabilized by an edge to face π–π interaction between the side chains of EVA-1 Phe-14 and CCL3 Phe-13 (inset). It appears that the insertion of an additional residue within the CC motif, as found in CXC chemokines, cannot readily be accommodated while retaining these key interactions.

Dias and coworkers also solved the crystal structure of CCL3 bound to EVA-1 with 1:1 stoichiometry (Figure 3A) [43]. CCL3 binds to the concave surface of EVA-1 created by the N-terminal region, the first β-sheet, and the C-terminal region. Since this region also forms the oligomerization interface within the EVA-1 dimer and trimer structures (Figure 2C), dimer dissociation may be required for chemokine binding, possibly explaining the slow binding kinetics observed for some Evasin:chemokine interactions [42].

The regions of CCL3 that interact with EVA-1 are the same regions required for binding and activation of chemokine receptors, providing a clear rationale for the ability of Evasins to inhibit chemokine function. Chemokines interact with their receptors through two main regions (Figure 3B). Initially, a shallow cleft defined by the so-called ‘N-loop’ and the third β-strand of the chemokine binds to the flexible N-terminal region of the receptor. Subsequently, the flexible N terminus of the chemokine inserts within the transmembrane helical bundle of the receptor, causing a conformational change and receptor activation 44, 45, 46, 47. EVA-1 binds to both of the critical functional regions of CCL3 (Figure 3A) [37]. The N-terminal region of EVA-1 interacts with the N-loop/β3 cleft of CCL3, thus mimicking the N-terminal regions of chemokine receptors. The N-terminal region of CCL3 binds to the first β-strand and the C-terminal region of EVA-1.

With the increased number of Evasin sequences available, it is now possible to identify details of the EVA-1:CCL3 complex that are likely to be conserved for other Evasins and chemokines. The selectivity of Class A Evasins for CC chemokines can be rationalized by considering the detailed interactions of CCL3 Cys-11 and Cys-12, as well as their disulfide bond partners (Cys-35 and Cys-51, respectively), with residues in EVA-1 (Figure 3C). It will be interesting to see whether specific Evasins contain variations of these interactions that enable CXC chemokine binding.

In addition to the above conserved features, the structure of EVA-1 bound to CCL3 also reveals interactions that are likely to be specific to this complex [37]. In particular, the N-terminal region of EVA-1 interacts with nonconserved residues in the N-loop of CCL3 and the C-terminal region of EVA-1 interacts with nonconserved residues in the N terminus of CCL3.

## Class B Evasins

### Protein Sequence Features

**Figure 4:**
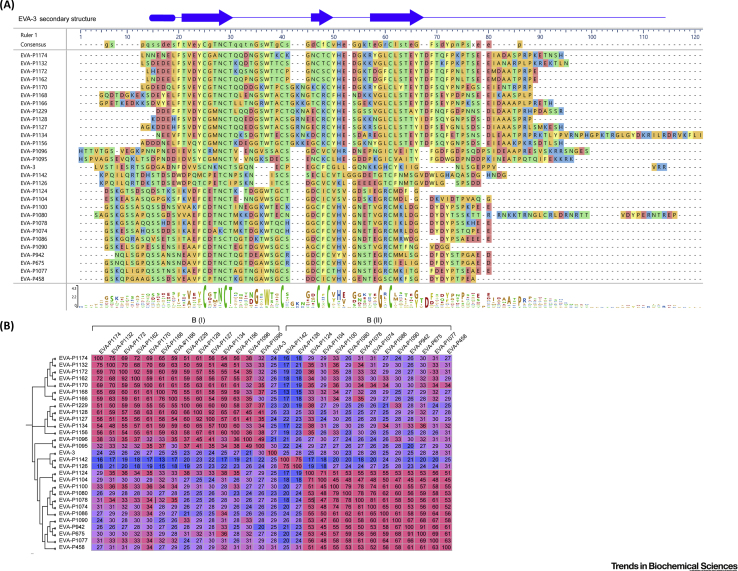
Sequence Alignments, Phylogenetic Tree, and Pairwise Identity Matrix of Class B Evasins. (A) Sequence alignment of all validated Class B Evasins. The consensus sequence (above alignment) and sequence logo (below alignment) show that six cysteine residues (green) and one glycine residue are completely conserved across the family. The secondary structure of EVA-3 [Protein Database (PDB) ID: 6I31] is presented at the top. The alignment was performed using MAFFT, with default parameters, in the program DNASTAR Navigator 15 (DNASTAR, Madison, USA). Amino acid residues are color coded by physicochemical properties (aromatic, light yellow; acidic, medium salmon; basic, medium blue; nonpolar aliphatic, medium orange; polar neutral, medium green). (B) Phylogenetic tree (left side) and pairwise identity matrix of all validated Class B Evasins, indicating subclasses B(I) and B(II). Pairwise identities between sequences were calculated using MAFFT and are color coded on a continuous scale from rose (high identity) to blue (low identity). The phylogenetic tree was generated on FigTree v1.4.3 (http://tree.bio.ed.ac.uk/software/figtree/) using the alignment data from MAFFT.

The 29 validated Class B Evasins [39] are listed in Table S1b (see the supplemental information online) and their sequences (61–104 amino acid residues) are aligned in Figure 4A. The pairwise identity matrix (Figure 4B) clearly shows that these proteins can be subclassified into two distinct groups, with typically ∼30%–90% identity between pairs of proteins in the same group and ∼20–35% identity between pairs in different groups. Lee *at al*. [39] named these Classes I and II, but we use the nomenclature Class B(I) and B(II) to distinguish them from Class A Evasins. All Class B Evasins contain six completely conserved Cys residues and an absolutely conserved Gly, two residues before the final Cys. Class B Evasins contain predicted *O*- and *N*-glycosylation sites and are glycosylated when expressed in mammalian cells 8, 37, 39.

### Chemokine-Binding Properties

In stark contrast to the selectivity of Class A Evasins for CC chemokines, Class B Evasins bind selectively to CXC chemokines and exhibit no measurable binding to CC chemokines 8, 37, 39. The tightest binding reported is a *K*_d_ of 0.72 nM for binding of EVA-P1128 to CXCL1 [39]. As suggested for Class A Evasins and CC chemokines, the absolute CXC-chemokine selectivity of Class B Evasins suggests that they may recognize the CXC motif itself. Moreover, Class B(I) and B(II) Evasins exhibit distinct CXC chemokine selectivity profiles [39]. Class B(I) Evasins, exemplified by the class founder EVA-3, bind only to ELR^+^ CXC chemokines, which activate the neutrophil receptors CXCR1 and CXCR2 [24]. Class B(II) Evasins bind to both ELR^+^ and other CXC chemokines, such as CXCL10, 11, 12, and 13 [39]. Remarkably, Class B(II) Evasins do not bind the ELR^+^ chemokine CXCL8 [39].

### Structure of EVA-3

**Figure 5:**
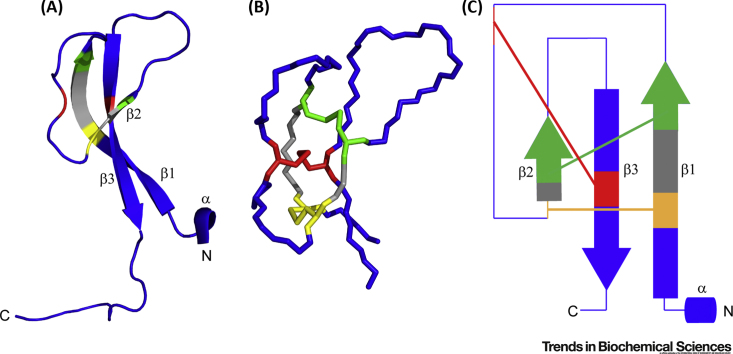
Structure of EVA-3 Showing the ‘Knottin’ Cystine Knot Topology. (A) Ribbon representation of one protomer of EVA-3 [Protein Database (PDB) ID: 6I31]. Pairs of cysteines that form disulfide bonds (Cys-21 and Cys-37; Cys-26 and Cys-39; Cys-33 and Cys-50) are shown in different colors (yellow, green, and red, respectively). Other residues within the macrocycle created by the first two disulfide bonds are shown in gray. (B) EVA-3 structure, colored as in (A), showing the protein backbone and Cys side chains. This view highlights the third (red) disulfide bond passing through the macrocycle created by the first two (yellow and green) disulfide bonds. (C) Topology diagram showing β-strands as arrows and the α-helix as a cylinder, with coloring corresponding to (A) and (B).

The structure of EVA-3 initially shown by Deruaz *et al.* [8] was refined as an asymmetric dimer containing a cystine knot [39]. The protomer structure was recently confirmed by NMR analysis [48]. Each protomer has a ‘**knottin**’ cystine knot topology (Figure 5A–C), consisting of a single layer β-sheet linked by two long loops, with these elements connected by three disulfide bonds, one of which passes through the macrocycle formed by the other two disulfides. The disulfide bond connectivity, but not the cystine knot topology, had been recently predicted using SecScan [49]. Based on the conservation of Cys residues and modelling studies [39], the knottin topology is expected to be retained across the Class B Evasins. Notably, the cystine knot is likely to increase the structural, proteolytic, and chemical stability of these proteins, enhancing their potential clinical utility [50]. Knottins have diverse biological activities ranging from protease inhibition to ion channel blockade [51]. The five loop segments in knottins mediate protein interactions and can be engineered for development as diagnostic and therapeutic agents 51, 52 such as ziconotide (Prialt®) and linaclotide (Linzess®). The Class B Evasins represent the first knottin family isolated from ticks. They are also the first chemokine-binding knottins reported.

### Structural Basis of Chemokine Recognition

In view of the lack, until very recently, of structural data for EVA-3 bound to a ligand, Lee *et al.* identified some chemokine recognition elements by exchanging sequences between EVA-3 and EVA-P1142, representatives of Class B(I) and Class B(II), respectively, with distinct chemokine selectivities [39]. Briefly, they found that several of the regions between the Cys residues contribute to chemokine binding selectivity. Structural modelling indicated that these regions are surface exposed and differ in both shape and charge properties. In particular, the ‘S5’ region (between Cys-5 and Cys-6) is highly positively charged in Class B(I) and neutral or negative in Class B(II), potentially contributing to the more restricted chemokine binding profiles of Class B(I) Evasins. Consistent with this proposal, the recent description of EVA-3 (truncated at both N and C termini) docked to CXCL8 [48] showed that the S5 region of EVA-3 interacts with the β1-strand of CXCL8, which explains why binding of EVA-3 disrupts dimerization (and the related glycosaminoglycan binding) of CXCL8. In addition, this docked structure indicated that the regions of EVA-3 close to the N terminus of the β1-strand and the C terminus of the β3-strand bind to a cavity between the α-helix and N-loop/helical turn of CXCL8. These interactions were accompanied by structural rearrangement of the chemokine, suggested to be responsible for inhibition of receptor binding.

## Outlook for Clinical Application of Evasins

The discovery of numerous Evasins with differing chemokine-binding selectivity, and their systemic antichemokine effects following parenteral administration, indicate that Evasins could be of therapeutic use in the treatment of chemokine-associated inflammatory diseases. To date, therapeutic intervention through blockade of chemokine activity by several different approaches has not been particularly successful 53, 54. This lack of success has often been attributed to the promiscuity of the system, in that several chemokines need to be neutralized to be efficacious in inflammation. It appears that natural selection in ticks has overcome this problem by devising molecular entities that are not highly selective, in contrast to small molecule receptor antagonists or antichemokine antibodies. As discussed above, the efficacy of EVA-1, -3, and -4 in animal models of disease supports the potential of these or other Evasins as clinical anti-inflammatory agents in humans.

However, immunogenicity is a key issue to be considered in therapeutic development. Ticks appear to have addressed this question as they produce Evasins as highly glycosylated proteins, which is predicted to considerably reduce their antigenicity. Whilst analysis of their T cell epitopes predicts that they are potentially less antigenic than human interferon-β [55], the immune response elicited by them will not be known until they are administered to humans. Concerns regarding immunogenicity could, however, be mitigated by targeted mutation of potential T cell epitopes in Evasins and/or by focusing translation of Evasins to acute indications where a single or short-term administration will likely prove effective. The successful use of the mouse neutrophil chemoattractant inhibitors EVA-3 in acute myocardial infarction [56] and in acute pancreatitis [32] and EVA-1 in acute lung injury [26] supports the exploration of an Evasin that inhibits neutrophil chemoattractants in human studies of these acute diseases. Other indications where chemokines have been validated as targets, and single or short-term Evasin therapy may be envisaged, include acute myocarditis [57] and acute stroke [58].

## Concluding Remarks

The studies reviewed here have revealed the existence of at least two classes of tick Evasins with inhibitory activity against mammalian chemokines. These recent discoveries raise a number of questions regarding the natural roles of Evasins and their potential for medical applications (see Outstanding Questions).

The expression of Evasins by diverse tick species suggests that they play important roles in promoting tick blood-feeding and survival. Considering that ticks are vectors for viral and bacterial pathogens in humans and both domestic and wild animals [59], it will be important to assess the contributions of Evasins to the spread of infectious diseases and to examine their potential as targets for tick control strategies such as vaccination.

Although hundreds of putative Evasins have been identified, only a few have been validated as chemokine binders. It is likely that many of the additional putative Evasins also inhibit chemokine activity. Furthermore, as additional tick genome and transcriptome sequences become available, many more Evasins are likely to be identified and new Evasin classes may even be discovered. Characterization of these proteins will help to establish the spectrum of chemokine selectivity across the Evasin family as well as providing clues about their biological functions and evolution. In particular, it will be interesting to explore whether Evasins have coevolved with host chemokines as ticks have adapted to different hosts.

Structure–function studies have begun to reveal the molecular basis of Evasin–chemokine recognition. However, much more work is needed to identify the features of Evasins contributing to their chemokine-binding affinity and selectivity. The insights obtained from such studies will assist in both the selection of natural Evasins that may target chemokines of interest and the rational or combinatorial engineering of Evasins for desired applications. Considering the effectiveness of Evasins in preclinical models of inflammatory diseases, there is a compelling incentive to further understand and develop this family of anti-inflammatory proteins.

Outstanding Questions

Are Evasins critical for tick infestation and potential targets for tick control?
How widespread and variable is the Evasin protein family?
Do additional classes of Evasins exist?
What is the structural basis of chemokine inhibition and selective CXC chemokine recognition by Class B Evasins?
What are the rules governing selectivity of Evasin-chemokine recognition?
How does the selectivity of Evasins vary for chemokines from different host species?
Can Evasins be engineered to tailor their target selectivity to a desired set of chemokines?
Can Evasins be engineered and formulated for effective therapeutic delivery to target tissues and sufficient pharmacokinetic stability?
Will Evasins be too immunogenic for therapeutic applications?

## Glossary

**Bioinformatics**: computational analysis of large biological data sets to identify novel, conserved, or unifying characteristics such as sequence or structural similarity.
**cDNA library**: set of complementary DNA (cDNA) sequences obtained by reverse transcription of mRNA from a biological sample of interest.
**Chemokines**: small proteins that are expressed at the sites of tissue injury and activate receptors on leukocytes, causing migration of the leukocytes to the injured tissues.
**CC chemokines**: a subfamily of the chemokine superfamily with the first two cysteine residues adjacent.
**CXC chemokines**: a subfamily of the chemokine superfamily with the first two cysteine residues separated by a single amino acid.
**Disulfide bond**: a covalent S–S bond between the side chains of cysteine residues in a protein, often stabilizing the folded structure; typically, the pattern of disulfide bonds is a conserved characteristic of a particular protein family.
**ELR**^**+**^**CXC chemokines**: the subset of CXC chemokines that contain the sequence Glu-Leu-Arg near their N termini.
**Evasins**: tick salivary glycoproteins that bind and inhibit host chemokines.
**Expression cloning**: a method in which a cDNA library and expression vector are used to generate a library of clones, each expressing a single protein, and then screened to identify the clones that express proteins with a property of interest (e.g., binding to a target).
**Glycosylation**: attachment of carbohydrate chains to proteins (*N*-linked via Asn side chains; or *O*-linked via Ser/Thr side chains); a heterogeneous post-translational modification that can affect various properties of the protein such as stability, solubility, immune recognition, and interactions.
**Inflammation**: the physiological response to tissue injury or infection.
**Inflammatory disease**: condition in which excessive or prolonged inflammation causes damage to the body’s cells or tissues.
**Knottin**: a protein structural motif (also known as ‘inhibitor cystine knot’) containing three disulfide bonds, one of which passes through the loop formed by the other two disulfides.
**Leukocytes**: any of several types of white blood cells, which play key roles in the body’s innate and adaptive immune responses.
**Mass spectrometry**: an analytical technique for determining molecular masses; commonly used for protein identification, validation, and characterization of post-translational modifications.
**Tick**: small (∼3–5 mm) hematophagous arachnid that parasitizes mammals, birds, and sometimes reptiles or amphibians.
**Yeast surface display**: expression of a library of proteins as fusions with a yeast cell wall protein, enabling the proteins to be displayed on the yeast cell surface for selection and isolation of those with desired binding properties.
