# Supplementary material for: Evasins: Tick Salivary Proteins that Inhibit Mammalian Chemokines
Source: Trends Biochem Sci. 2020 Feb;45(2):108–22. doi: 10.1016/j.tibs.2019.10.003 (PMC7322545; doi:10.1016/j.tibs.2019.10.003)
Supplement: Supplementary Material [file mmc1.docx]

**Supplementary information**

**Evasins: Tick Salivary Proteins that Inhibit Mammalian Chemokines**

Ram P. Bhusal^1^, James R.O. Eaton^2^, Sayeeda T. Chowdhury^1^, Christine A. Power^3^, Amanda E.I. Proudfoot^4^*, Martin J. Stone^1^* and Shoumo Bhattacharya^2,^*

^1^Infection and Immunity Program, Monash Biomedicine Discovery Institute, and Department of Biochemistry and Molecular Biology, Monash University, Clayton, VIC 3800, Australia.

^2^RDM Division of Cardiovascular Medicine and Wellcome Trust Centre for Human Genetics, University of Oxford, Roosevelt Drive, Oxford, OX3 7BN, United Kingdom.

^3^Biopharm Discovery, GlaxoSmithKline, Gunnels Wood Road, Stevenage, Hertfordshire SG1 2NY, United Kingdom.

^4^Novimmune SA, 14 chemin des Aulx, 1228 Plan les Ouates, Switzerland.

*Correspondence: amandapf@orange.fr (A.E.I. Proudfoot); martin.stone@monash.edu (M.J. Stone); sbhattac@well.ox.ac.uk (S. Bhattacharya).

**Table S1a**: Systematic Nomenclature for Class A Evasins

**Table S1b**: Systematic Nomenclature for Class B Evasins

**Table S1a**: Systematic Nomenclature for Class A Evasins

| **Systematic Name** | **Class** | **Tick Species^1^** | **Previous Name(s) and Reference(s)** |
| --- | --- | --- | --- |
| EVA-1 | A | *R. sanguineus* | Evasin-1 [1] |
| EVA-4 | A | *R. sanguineus* | Evasin-4 [2] |
| EVA-P467 | A | *R. pulchellus* | P467_RHIPU [3], RPU-01 [4] |
| EVA-P546 | A | *A. cajennense* | P546-AMBCA [3] |
| EVA-P672 | A | *R. pulchellus* | P672_RHIPU [5] |
| EVA-P974 | A | *A. cajennense* | P974-AMBCA [3], ACA-01 [4] |
| EVA-P983 | A | *A. cajennense* | P983-AMBCA [3] |
| EVA-P985 | A | *A. parvum* | P985-AMBPA [3], APA-01 [4] |
| EVA-P991 | A | *A. cajennense* | P991_AMBCA [3] |
| EVA-P1180 | A | *A. triste* | P1180-AMBTR [3] |
| EVA-P1181 | A | *A. maculatum* | P1181-AMBMA [3] |
| EVA-P1182 | A | *A. maculatum* | P1182-AMBMA [3] |
| EVA-P1183 | A | *A. triste* | P1183-AMBTR [3] |
| EVA-AAM-01 | A | *A. americanum* | AAM-01 [4] |
| EVA-AAM-02 | A | *A. americanum* | AAM-02 [4], P1243 [6] |
| EVA-ACA-02 | A | *A. cajennense* | ACA-02 [4] |
| EVA-AMA-01 | A | *A. maculatum* | AMA-01 [4] |
| EVA-ATR-02 | A | *A. triste* | ATR-02 [4] |
| EVA-IHO-01 | A | *I. holocyclus* | IHO-01 [4] |
| EVA-IRI-01 | A | *I. ricinus* | IRI-01 [4] |
| EVA-RPU-02 | A | *R. pulchellus* | RPU-02 [4] |

**Table S1b**: Systematic Nomenclature for Class B Evasins

| **Systematic Name** | **Class** | **Tick Species^1^** | **Previous Name(s) and Reference(s)** |
| --- | --- | --- | --- |
| EVA-3 | B | *R. sanguineus* | Evasin-3 [7], EVA3_RHISA [8] |
| EVA-P1174 | B | *I. ricinus* | P1174_IXORI [8] |
| EVA-P1170 | B | *I. ricinus* | P1170_IXORI [8] |
| EVA-P1132 | B | *I. ricinus* | P1132_IXORI [8] |
| EVA-P1172 | B | *I. ricinus* | P1172_IXORI [8] |
| EVA-P1162 | B | *I. ricinus* | P1162_IXORI [8] |
| EVA-P1168 | B | *I. ricinus* | P1168_IXORI [8] |
| EVA-P1166 | B | *I. ricinus* | P1166_IXORI [8] |
| EVA-P1229 | B | *I. ricinus* | P1229_IXORI [8] |
| EVA-P1156 | B | *I. ricinus* | P1156_IXORI [8] |
| EVA-P1128 | B | *I. ricinus* | P1128_IXORI [8] |
| EVA-P1127 | B | *I. ricinus* | P1127_IXORI [8] |
| EVA-P1134 | B | *I. ricinus* | P1134_IXORI [8] |
| EVA-P1096 | B | *I. ricinus* | P1096_IXORI [8] |
| EVA-P1095 | B | *I. ricinus* | P1095_IXORI [8] |
| EVA-P1142 | B | *A. cajennense* | P1142_AMBCA [8] |
| EVA-P1126 | B | *A. cajennense* | P1126_AMBCA [8] |
| EVA-P1124 | B | *I. ricinus* | P1124_IXORI [8] |
| EVA-P1104 | B | *I. ricinus* | P1104_IXORI [8] |
| EVA-P1100 | B | *I. ricinus* | P1100_IXORI [8] |
| EVA-P1080 | B | *I. ricinus* | P1080_IXORI [8] |
| EVA-P1078 | B | *I. ricinus* | P1078_IXORI [8] |
| EVA-P1074 | B | *I. ricinus* | P1074_IXORI [8] |
| EVA-P1090 | B | *I. ricinus* | P1090_IXORI [8] |
| EVA-P942 | B | *I. ricinus* | P942_IXORI [8] |
| EVA-P675 | B | *I. ricinus* | P675_IXORI [8] |
| EVA-P1086 | B | *I. ricinus* | P1086_IXORI [8] |
| EVA-P1077 | B | *I. ricinus* | P1077_IXORI [8] |
| EVA-P458 | B | *I. ricinus* | P458_IXORI [8] |

^1^*R, Rhipicephalus; A, Amblyomma; I, Ixodes*.

**Table S1 References**

1. Frauenschuh, A. et al. (2007) Molecular cloning and characterization of a highly selective chemokine-binding protein from the tick Rhipicephalus sanguineus. *J. Biol. Chem.* 282 (37), 27250-8.

2. Déruaz, M. et al. (2013) Evasin‐4, a tick‐derived chemokine‐binding protein with broad selectivity can be modified for use in preclinical disease models. *FEBS J.* 280 (19), 4876-4887.

3. Singh, K. et al. (2017) Yeast surface display identifies a family of evasins from ticks with novel polyvalent CC chemokine-binding activities. *Sci. Rep.* 7 (1), 4267.

4. Hayward, J. et al. (2017) Ticks from diverse genera encode chemokine-inhibitory evasin proteins. *J. Biol. Chem.* 292 (38), 15670-15680.

5. Eaton, J.R. et al. (2018) The N-terminal domain of a tick evasin is critical for chemokine binding and neutralization and confers specific binding activity to other evasins. *J. Biol. Chem.*, jbc. RA117. 000487.

6. Alenazi, Y. et al. (2018) Genetically engineered two-warhead evasins provide a method to achieve precision targeting of disease-relevant chemokine subsets. *Sci. Rep.* 8 (1), 6333.

7. Deruaz, M. et al. (2008) Ticks produce highly selective chemokine binding proteins with antiinflammatory activity. *J. Exp. Med.* 205 (9), 2019-31.

8. Lee, A.W. et al. (2019) A knottin scaffold directs the CXC-chemokine-binding specificity of tick evasins. *J. Biol. Chem.*
